# Supplementary figures and images for: Domain-adaptive neural networks improve supervised machine learning based on simulated population genetic data
Source: PLoS Genet. 2023 Nov 7;19(11):e1011032. doi: 10.1371/journal.pgen.1011032 (PMC10655966; doi:10.1371/journal.pgen.1011032)

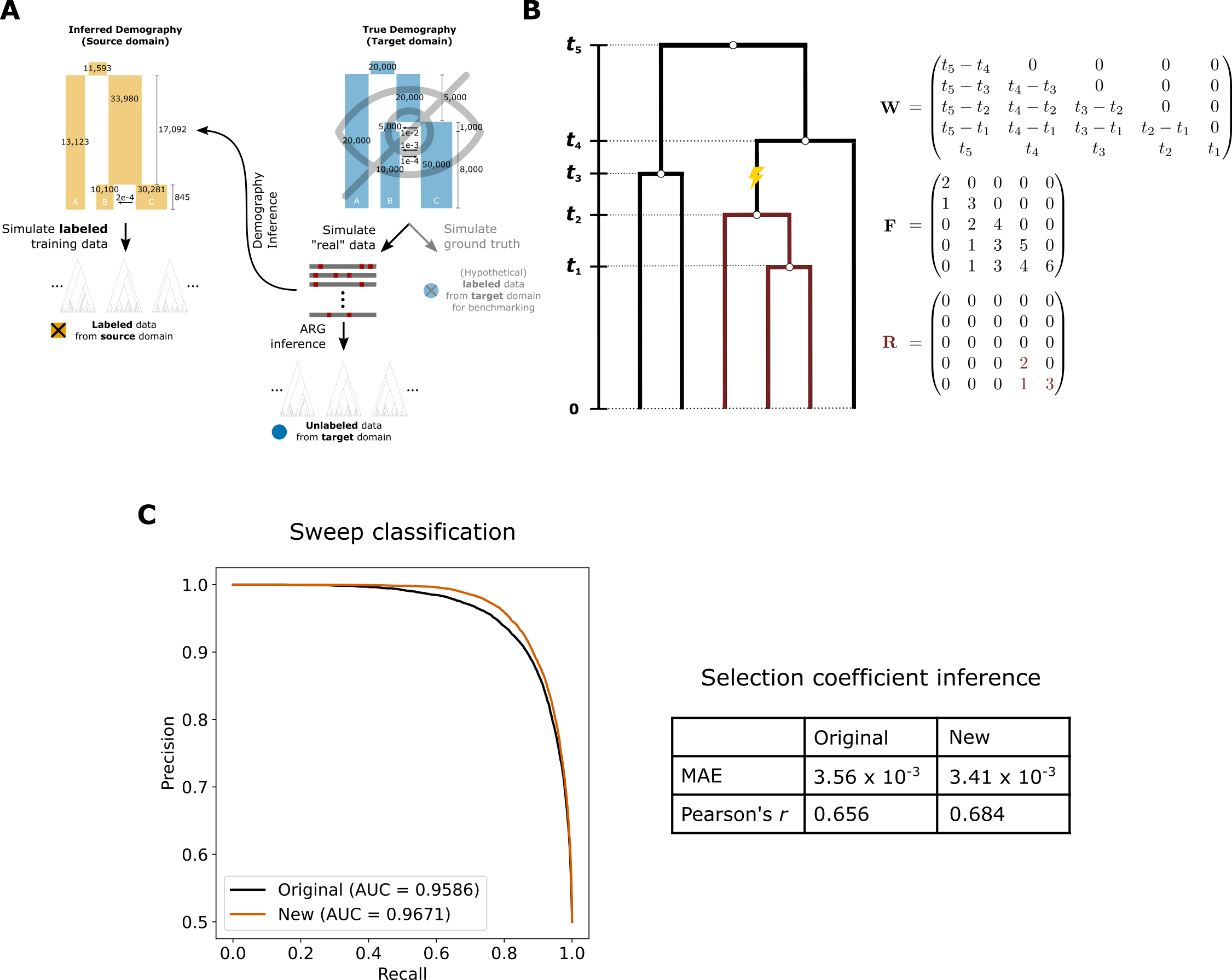

Supplement: S1 Fig — A) The workflow of a simulation study that aims to benchmark the performance of the domain-adaptive SIA model in a realistic setting of demographic mis-specification. B) An improved version of SIA input features that encodes the full genealogy (adapted from [59]). A genealogy with n taxa at a polymorphic site is uniquely encoded by three (n-1) x (n-1) lower triangular matrices. The weight matrix W encodes the coalescent intervals where wij = tn−j−tn−1−i, ∀i≥j, and the topology matrix F encodes the number of lineages persistent in the coalescent intervals corresponding to W (i.e. fij = # of lineages between tn−j and tn−1−i, ∀i≥j). The derived lineage matrix R encodes only the subtree subtending the branch where the mutation occurred (red lightning symbol), following the same scheme as F. Note that the W matrix is a redundant encoding of the n-1 coalescent times (t1,t2, …,tn-1), which contains information roughly equivalent to the original SIA input features [12]. C) Comparison of the performance of the new SIA input features in (B) to that of the original SIA input features. (TIF) [file pgen.1011032.s001.tif]

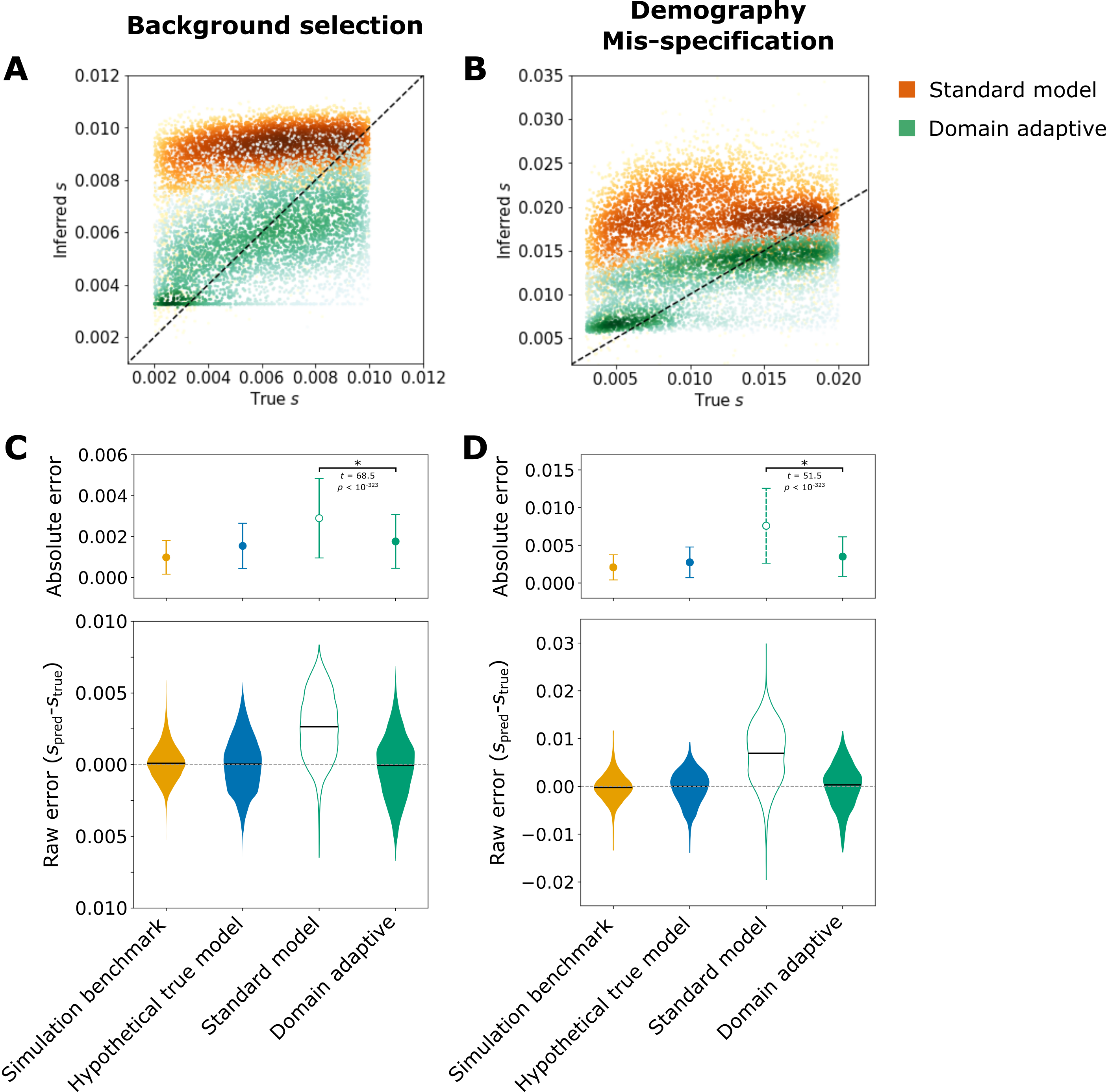

Supplement: S2 Fig — Raw data used to plot Fig 3B and 3D are presented in (A) and (B), respectively. Performance of SIA models in the simulation experiment of failure to account for background selection (C) and in the simulation experiment of demographic model mis-specification (D) is presented in terms of mean and standard deviation of the absolute error (top) as well as the distribution of raw error (bottom). Statistical significance (*) of the difference between the absolute error of the standard model and that of the domain-adaptive model is evaluated with Welch’s t-test. See Fig 1C for definition of the model labels. (TIF) [file pgen.1011032.s002.tif]

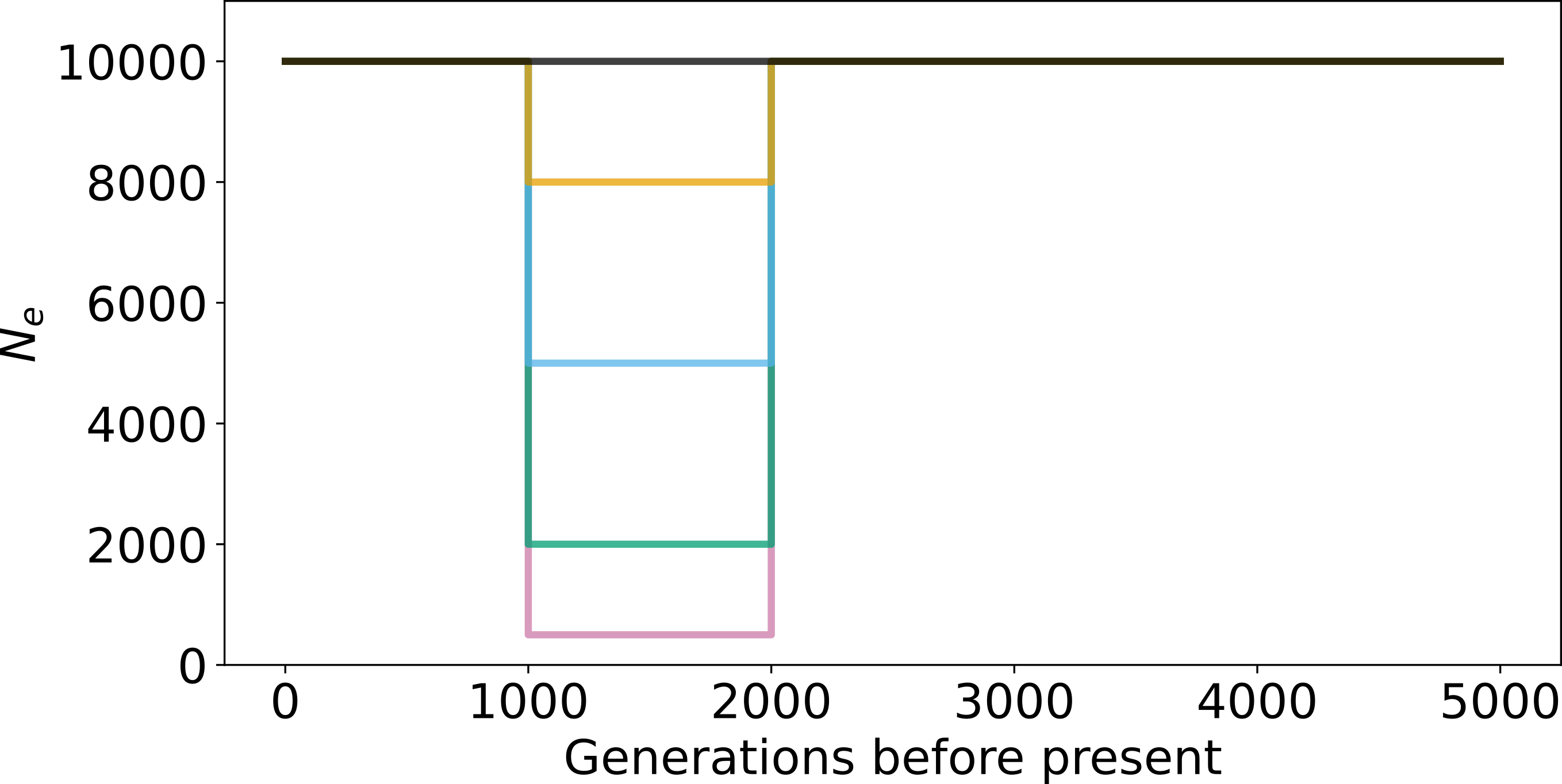

Supplement: S4 Fig — (TIF) [file pgen.1011032.s004.tif]

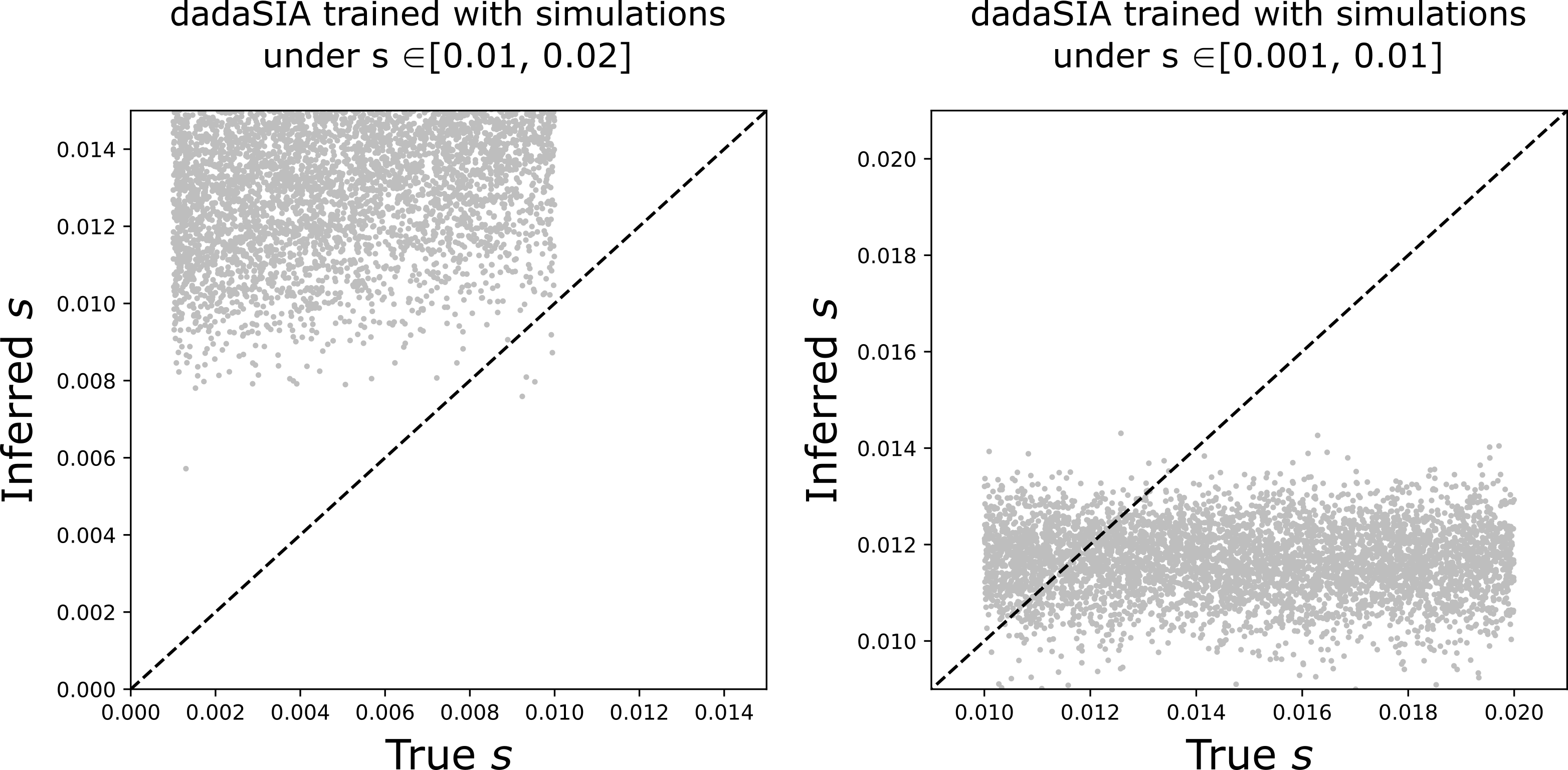

Supplement: S5 Fig — The dadaSIA model trained with source domain data under s∈[0.01, 0.02] failed to meaningfully infer any value lower than 0.01, even when examples of s∈[0.001, 0.01] were supplied to the model as “unlabeled” target domain data, and vice versa. (TIF) [file pgen.1011032.s005.tif]

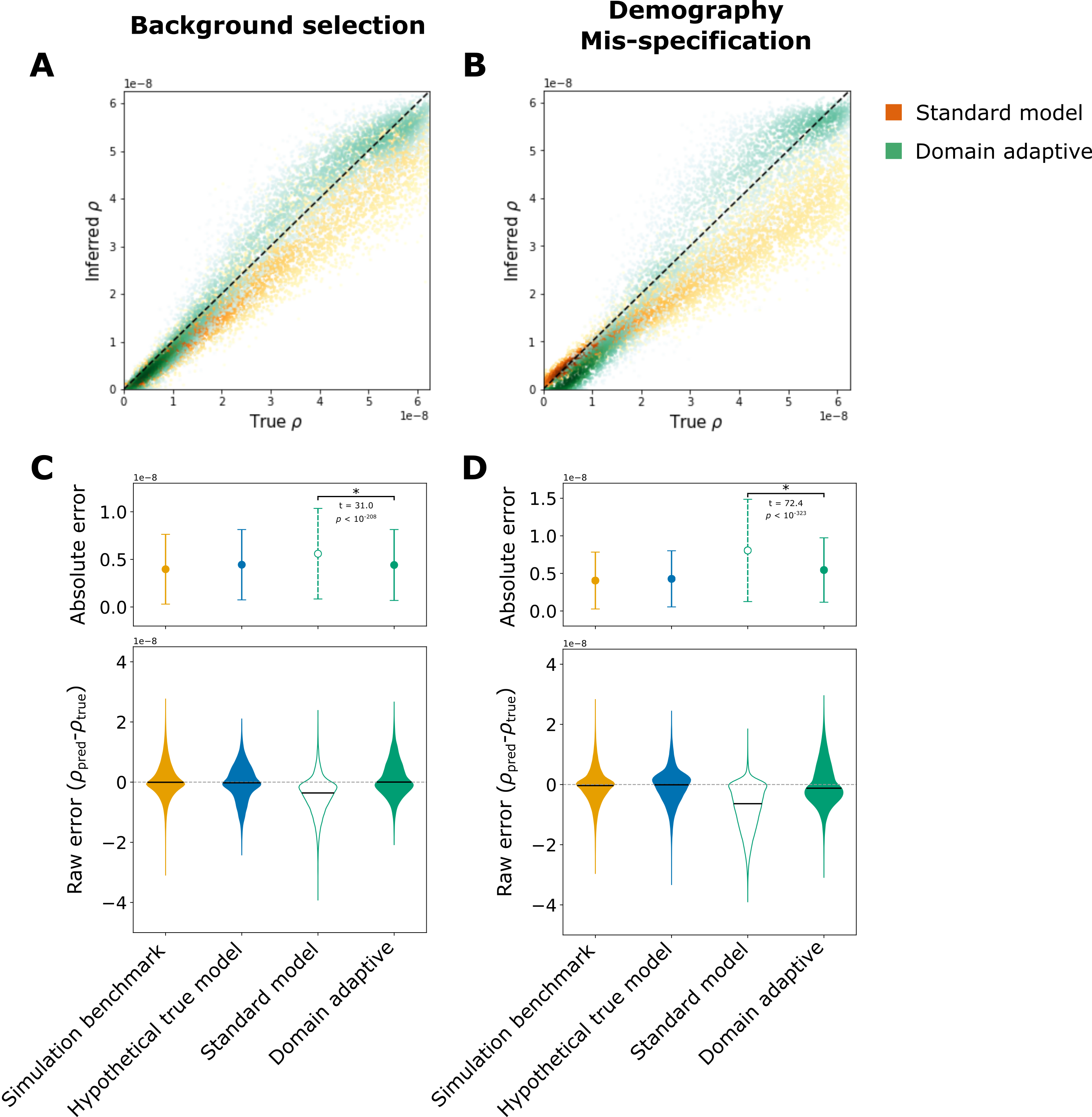

Supplement: S6 Fig — Raw data used to plot Fig 4A and 4B are presented in (A) and (B), respectively. Performance of ReLERNN models in the simulation experiment of failure to account for background selection (C) and in the simulation experiment of demographic model mis-specification (D) is presented in terms of mean and standard deviation of the absolute error (top) as well as the distribution of raw error (bottom). Statistical significance (*) of the difference between the absolute error of the standard model and that of the domain-adaptive model is evaluated with Welch’s t-test. See Fig 1C for definition of the model labels. (TIF) [file pgen.1011032.s006.tif]

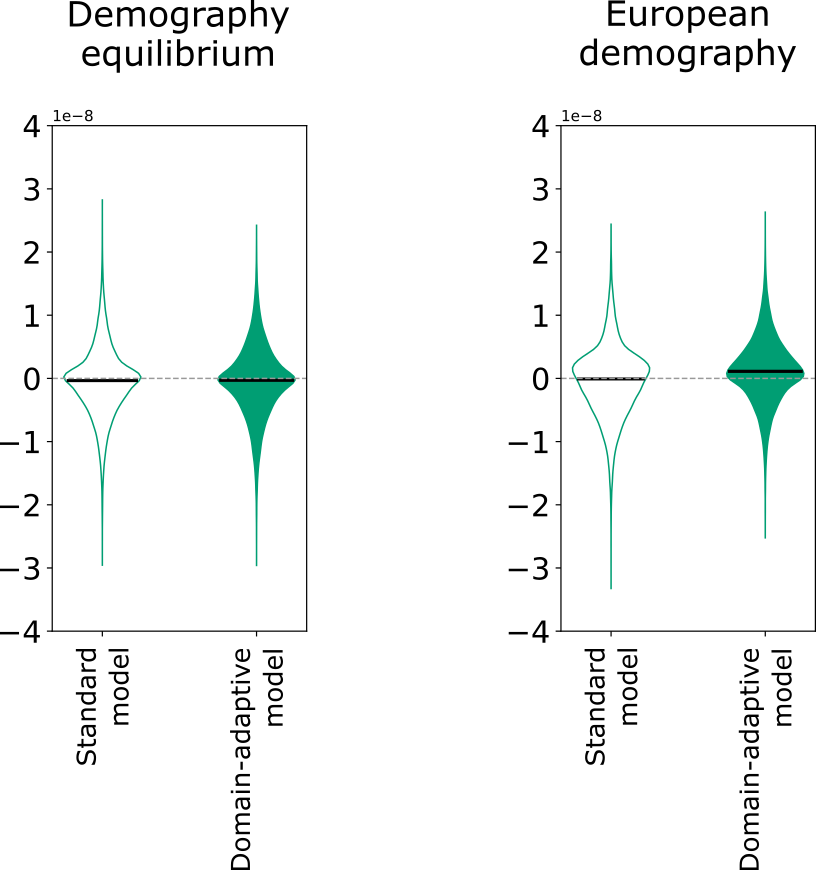

Supplement: S7 Fig — The respective mean absolute error (MAE) of the standard and domain-adaptive models are 4.05 x 10^−9^ and 4.13 x 10^−9^, under demography equilibrium, and 4.28 x 10^−9^ and 3.93 x 10^−9^, under a European demography. Note that the domain-adaptive model has a slight upward bias in its estimates in the case of European demography. (TIF) [file pgen.1011032.s007.tif]

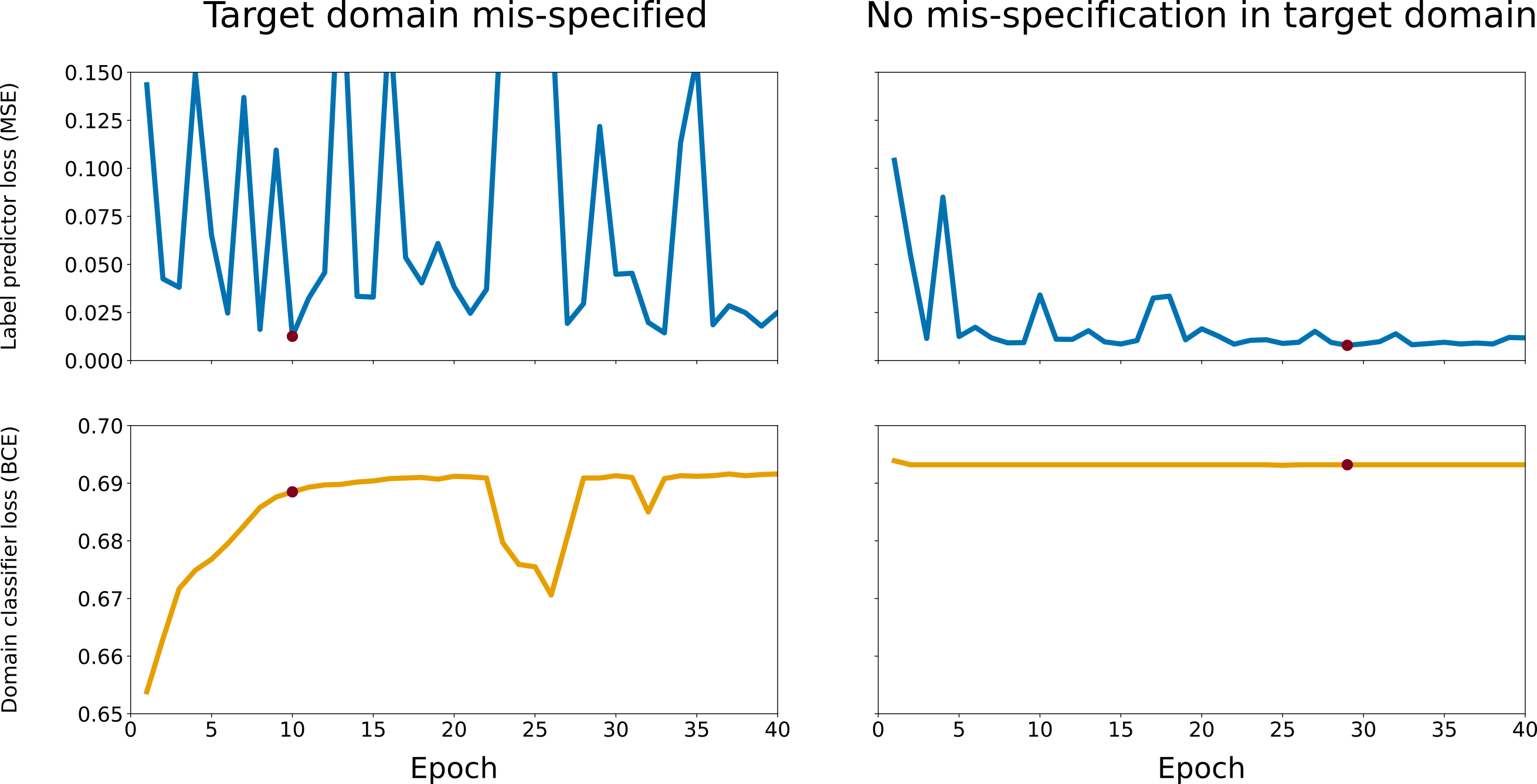

Supplement: S8 Fig — The losses of the domain-adaptive ReLERNN models during training are plotted with and without simulation mis-specification. The red dot marks the early-stopping epoch (i.e. epoch with the lowest validation loss for the label predictor). (TIF) [file pgen.1011032.s008.tif]

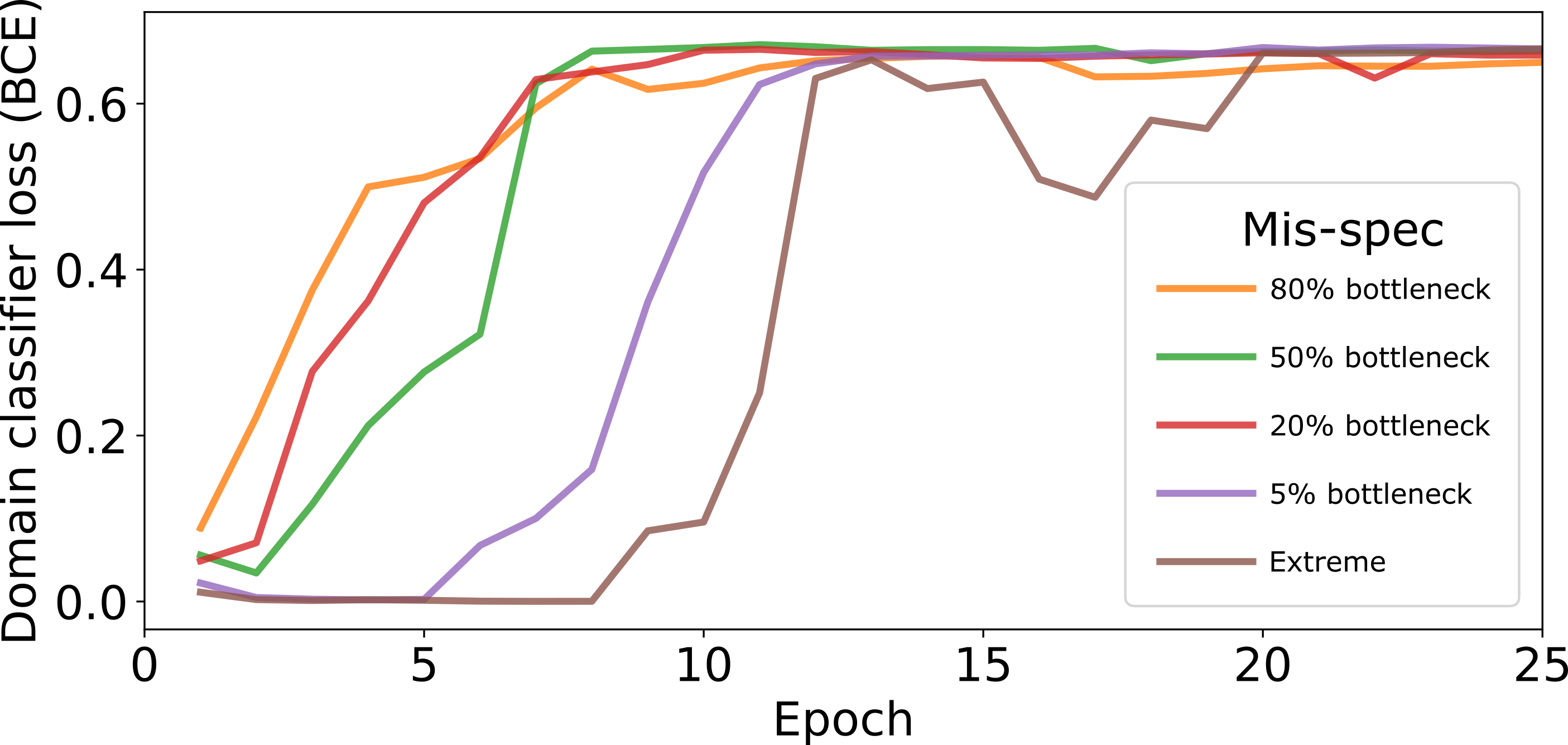

Supplement: S9 Fig — See Fig 5 and Methods for details of the types of mis-specification. (TIF) [file pgen.1011032.s009.tif]
