# Supplementary material for: Domain-adaptive neural networks improve supervised machine learning based on simulated population genetic data
Source: PLoS Genet. 2023 Nov 7;19(11):e1011032. doi: 10.1371/journal.pgen.1011032 (PMC10655966; doi:10.1371/journal.pgen.1011032)
Supplement: Attachment — Submitted filename: mo_and_siepel_review.pdf [file pgen.1011032.s010.pdf]

In their study, Mo and Siepel show the impact of model mis-specification on the performance of simulation-based supervised deep learning methods in population genetics and propose an approach to mitigate the issue. More precisely, they rely on an adversarial domain-adaptation (DA) technique proposed in the machine learning field and integrate it into two different methods, (i) a selection inference tool SIA, previously published by their lab ; (ii) a recombination inference tool, RELERNN by Adrion and Kern. They convincingly show that domain adaptation can “rescue” a method that made otherwise poor and/or biased predictions.

Using two tools has multiple interests: (a) showing the versatility of the domain-adaptation technique that can be somehow “plugged” into different network architectures, for different tasks (estimating selection versus recombination), with different levels of expert knowledge regarding the original method (one is developed by their lab, one not) ; (b) investigating the effect of mis-specification and domain adaptation in different scenarios, where mis-specified “nuisance” parameters can have more or less impact on the performance ; (c) discuss the impact of tree reconstruction (used for SIA but not for RELERNN), but I will go back to this point in my comments.

Although I have multiple comments that I would like to see addressed for clarification or because I think they could help understand the applicability of the approach in certain conditions (eg little mis-specification, small target domain size), Mo and Siepel’s study is timely, thorough and overall well described. I thus think that, provided my comments are addressed, the manuscript should be accepted: it will be of wide interest to plos genetics readers, both methodology developers who could quickly try integrating DA into their tools, and users since it highlights clearly the impact of mis-specification (a general concern in the population genetics field) and explains the principle and additional value of DA in a way that should be understandable by many (with helpful figures).

Major comments

Q1 The methodology underlying the “simulation benchmark” and “hypothetical model” should be stated more clearly. There might be confusion for the reader because it could be either (a) the original methods (SIA or RELERNN) trained and applied to the same dataset (in one case the “simplistic” simulations, in the other the hypothetical true - more complex – model); or (b) their domain adaptive version (with matching source and target domains). I understood it is (a), but it was not crystal clear at first read. Here is the sentence that I find ambiguous:

l.161 For additional context, we also considered the two cases where the training and testing domains matched (source-matched or target-matched; Fig. 1C)—although we note that these cases are not achievable with real data and provide only hypothetical upper bounds on performance.

Could the author clarify the sentence / the schematic table of Fig. 1 (add a short description in methods?)?

I actually see value in doing both experiments:

(a) Training and applying the regular method to matching domains has to be done since it provides the best possible performances (for simple simulations on one side, for hypothetical true model on the other). Compared to dadaSIA, it allows understanding if there is room for future improvements since, as the authors discussed l. 321, other domain adaptation techniques exist.

(b) Comparing (a) to the domain adaptive versions, still with matching domains, allows evaluating how much DA decreases (or not) the performances in a case where we would not have needed it. In this case, it can help understand the model's behavior: Is the discriminator not learning anything, hence not influencing the computed features? Or is it somehow overfitting and giving unwanted feedback to the feature computation branch? The authors could also display training and validation losses of the different branches through training.

Of course, in real life it is complicated to know whether mis-specification is present (we can argue that it is very likely the case given that "all models are wrong"), and I agree with the statement that it is not achievable, so even if DA decreases performances in case (b), the method should still be valuable (but see Q2).

Q2 Related to Q1, if one observes a decrease in performances in (b), it would be helpful to understand what level of mis-specification is "required" for DA to start being valuable (i.e. to start increasing performance compared to the non DA standard application case). To design such an experiment, one needs target datasets that get increasingly divergent from the source with small incremental steps. For example, it could be the background selection experiment with a purifying selection coefficient going gradually away from zero. I am aware that it is a costly experiment as it requires a new training for each step, but I believe the answer informative to know to what extent researchers should all move to using DA in neural networks, or if this has a hidden risk.

Q3 The target domain size is currently the same as the training size, i.e. very large. I am not certain it is a reasonable assumption, given that the target domain corresponds to the data, which in many cases is more limited than simulations. For example, in the real application of SIA the target size is 1 million; however, those are not independent examples (contrary to the simulated target domains). ReLERNN was not applied to real data, but I understand that we would expect to have fewer target examples, since one example does not consist of a single tree but of a long genotype matrix. So would DA work as it is currently set up? This could influence the strategy chosen for the loss weight (l.347-348).
The authors should comment on those points and, if they agree that size is often limited for real data, evaluate the impact of source-to-target size ratio / discuss based on previous ML literature, as this might limit the use of DA in practice.
I note that the authors provide an interesting related experiment where the labels are imbalanced in the target domain but not in the source domain. It is also very relevant in terms of benchmarking for a realistic target domain, hence for applications to real data, but answers a different question.

Q4 The discussion around the impact of tree reconstruction versus other nuisance parameters (demography/background selection) is interesting, especially given the recent trend of relying more

and more on reconstructed trees and extending tree inference tools to other tasks. However, I am not fully convinced that the following statement is completely backed up:

l.285 *For example, the performance gap between the "simulation benchmark" (trained and tested on simulated data) and "hypothetical true" (trained and tested on real data) models was considerably greater for SIA than for ReLERNN (Figs. S2C&D, S4C&D). This difference appears to be driven by ARG inference, which is required by SIA in the hypothetical true case but not the simulation benchmark case, and for which no analog exists for ReLERNN.*

Indeed it is possible that the demography and selection mis-specifications have way more impact on SIA than on RELERNN, regardless of tree reconstruction, leading to the qualitative differences in the usefulness of DA for the two tasks.
A perfect experiment would be to include NO mis-specification for these nuisance parameters (demo or selection) but only for the tree reconstruction (source with true trees, target with reconstructed trees) and compare performances without and with DA. Was this done? If yes, please clarify the text. If not, the authors should temper their conclusion or, more interestingly, do the experiment. This experiment would be useful to many researchers developing inference tools based on reconstructed ARGs.
Going further, but likely for future work, one wonders if DA is better to fix the gap than reconstructing the trees for all simulated data so that both source and real target domain are based on inferred trees. Even if slightly worse, the computational gain of using DA and not inferring all trees could be valuable.

Important:
The discussion could be extended to reply to the following questions Q5-8:
Q5 Could the authors discuss the impact of prior mis-specification for target parameters (such as real selection coefficient or recombination rate out of range) rather than for nuisance parameters (demo etc). I foresee that DA will fail in such a case.

Q6 Model mis-specification also strongly impacts non deep learning approaches (such as ABC on summary statistics) and even non-simulation-based approaches (model-based likelihood with simplifying assumptions). It could be good to discuss this, and I would enjoy reading the authors' view on whether DL approaches are the only ones that could benefit from such domain adaptation techniques.

Q7 Although many previous works discussed model mis-specification and its impact on their methods by setting up robustness experiments (to different demographic models, to selection instead of neutrality, etc.) extremely few tested a solution, which is why this study is very valuable. One could discuss the papers that intentionally widen the models/priors to integrate more nuisance parameters, simulate errors in the data, or aim at co-estimating multiple processes (e.g. demo + selection). But the work closest to this study in terms of mis-specification mitigation is the one on automated inference using gan (cited by the authors). Hence it could be good to discuss a few resemblances and differences / advantages between the approaches. Some are clear to me (for example, the current approach is very flexible and integrable to most of the existing neural networks

for population genetics inference, and likely lighter computationally – but see a question below) but maybe less clear to a regular reader. I might also be missing some disadvantages.

Q8 In their previous paper (SIA Hejase et al. 2022), the authors did multiple experiments to test the impact of ARG inference and demography mis-specification. Could the authors put them in perspective with the experiment and findings of the current study? For example: (i) Are the levels of mis-specification and the yielded estimation errors similar between the two studies? (ii) Although the classification is reasonable, the coefficient estimates of SIA standard application (Figures 3&S2) are really poor (ie they do not seem usable in practice). It is a bit worrisome for papers using the original tool, as the mis-specification scenarios are very realistic. Can Hejase et al (2022) provide insight on how CLUES and imagene or other methods would perform in these scenarios?

Other comments:

- Are the comparisons always done using the improved input features (cf Fig S1)? Otherwise, I am worried that differences might not reflect (only) the introduction of DA but also the change of input representation. This question applies to benchmark experiments and the real data application (Table 1).

- What is the cost of training domain adaptive networks? How does it compare to the original versions?

- It would be great to give, in Sup Mat, training and validation losses (decomposed along branches) to get an insight on the model training behavior.

- Related, is there a way to leverage the gradient or losses information to approximate how mis-specified a model was? Losses with adversarial training can get tricky to evaluate, so this might not be trivial.

- l. 89 except maybe for ABC-RF

- Fig2 in the original SIA paper, the architecture is depicted as an RNN (LSTM) with no mention of convolutions, here there are only mentions of convolutional blocks. Please clarify whether some changes were made.

- l. 150- 153: it's a good way of setting the mis-specification. It would be useful to summarize in the text the main resemblances/differences: ie the model still consists of the same number of populations with constant population size in between split events and migration only between B and C, but the split between B and C is older followed by higher rates of migration compared to the source model.

- *l. 178 "In our experiments, the ARG is "known" (fixed in simulation) in this case, whereas in the hypothetical true model it must be inferred. Thus, the difference between these two cases represents a rough measure of the importance of ARG inference error (see Discussion)."*

and l.285 *For example, the performance gap between the "simulation benchmark" (trained and tested on simulated data) and "hypothetical true" (trained and tested on real data) models was considerably greater for SIA than for ReLERNN (Figs. S2C&D, S4C&D). This difference appears to be driven by ARG inference, which is required by SIA in the hypothetical true case but not the simulation benchmark case, and for which no analog exists for ReLERNN.*

I understand that (for SIA) for simple simulations, both source and target the true tree is used, and for hypothetical true, both source and target, the tree is inferred, whatever the experiment. Can the author clarify this in l178 and Method. It would **be very helpful** to have the equivalent of the schematic table of figure 1 for the SIA case, where the authors would add in each cell if trees are exact or inferred (possibly in sup mat).

I think that when the trees are inferred in both source and target, it is not a case model mis-specification but corresponds instead to noisy input data. Alternatively, when the true trees are used for the source and the inferred ones for the target, I agree it can be depicted as a type of model mis-specification.

- l.187 Imbalanced classes experiment. Can the author clarify whether the error is computed on a balanced test set or on a similarly imbalanced test set for each percentage? Could it also make sense to show the standard model error on each differently balanced test set?
What is the expected imbalance in, say, humans and other well-studied species?

- l.298 Fig 4,S4: Do the authors have an intuition regarding the underestimation of high recombination rates without DA, and the underestimation of low rates with DA?

- l. 241: *The seven loci predicted by SIA to be sweeps were also predicted by dadaSIA to be sweeps (Table 1), although dadaSIA always reported higher confidence in these predictions (...)*
Was this phenomenon of increased confidence observed in the previous experiments?

- *l.254 Together, these observations suggest that the addition of domain adaptation does not radically alter SIA's predictions for real data but* ***may in some cases improve them.***
The last part is a bit of a gamble because SIA was previously shown to be better than other methods (although I do not know if it was compared to all the methods used for the last column of Table 1), so going away from SIA estimates towards the literature's estimates might not be a good thing. Unless SIA with mis-specification is worst than previous methods? I believe the authors do not discuss this.

- l. 343 and 345: State the exact losses (MSE and binary cross entropy ? Penalization ?)

- l.349-361: It is not clear from this paragraph whether trees are inferred in some cases of the background selection experiment. It seems that not, but l.178 seems to say otherwise. Can you clarify? Or is it that l. 374-383 correspond to a methodology that applies to both types of mis-specification and should have its own title? Then l 384-390 rather fit in the "Demography mis-specification experiment with SIA" part, since demography inference is not done for the background selection experiment? Please clarify.

- in l 374-383. Can the author specify whether only the central tree of each simulated segment for pop B will be encoded and used to train SIA and dadaSIA for readers unfamiliar with SIA?

- l.388 I did not find the G-PhoCS file

- Method: The author should provide their code for the different simulation scenarios

- l.420 indicates the split training/validation/test for RELERNN; is it similar for SIA?
