# Supplementary material for: Domain-adaptive neural networks improve supervised machine learning based on simulated population genetic data
Source: PLoS Genet. 2023 Nov 7;19(11):e1011032. doi: 10.1371/journal.pgen.1011032 (PMC10655966; doi:10.1371/journal.pgen.1011032)
Supplement: Attachment — Submitted filename: response-to-comments.docx [file pgen.1011032.s011.docx]

# Editors’ comments

Three reviewers have each given thorough and insightful critiques of this manuscript. I have also read this paper carefully and discussed it with many colleagues, and I am in close agreement with the reviewers' recommendations. I think this paper represents a potentially important step forward in the design and application of deep learning methods for population genetic inference, but additional analyses recommended by the reviewers would strengthen the paper considerably. I think the reviewers have made some excellent suggestions and I would like to see the authors address them in a revision.

We would like to thank the editor and the reviewers for their interest in and attention to our work. They have all made thoughtful comments and suggestions that inspired several additional analyses and updates to rhetorical points that we believe have significantly strengthened the narrative.

I particular, I would like to stress the following points:

1) Reviewer #3 raises an important point that I think the authors should clarify: is misspecification a problem unique to machine learning methods, or a more universal problem in population genetic inference that machine learning methods may be especially useful for mitigating? I of course lean towards the second one here, and it is fine if the authors don't agree, but I would like them to share their thoughts on this.

We agree that mis-specification is a general problem in popgen inference beyond just supervised machine learning methods and we thank the editor and reviewer #3 for giving us the opportunity to discuss this (**l. 456-468**). As we now discuss, however, we think that the mis-specification plaguing simulation-based ML methods is slightly different from that affecting unsupervised, model-based methods and they call for different solutions.

2) I agree with the reviewers that pushing the misspecification boundary until things break would be a great, and probably essential, addition to this paper. Does domain adaptation ever hurt performance if the degree of misspecification gets to be too large (or too small)?

This is an excellent point and we agree with the editor and all three reviewers that it is important to demonstrate the capabilities of domain adaptation under a range of mis-specifications. To this end, we added a subsection titled “Efficacy of Domain Adaptation under Various Degrees of Simulation Mis-specification” in **Results** (**l. 263-289**) and discussed the implications of the results in **l. 370-397**. The main result is summarized in **Figure 5**.

3) As noted by the reviewers, the correct interpretation of the authors' comparison to previous selection coefficients is unclear. Why should we expect that the previous non-SIA estimates would be unaffected by model misspecification? If I recall, some of these estimates may have been based on partial allele frequency trajectories inferred from ancient DNA, but even estimates of s obtained from population genetic time-series data will be biased by misspecification. I therefore urge the authors to clarify what they think the take-home from this comparison is.

We have streamlined the presentation of the selection coefficient estimates in the **Results** to present just the findings (**l. 305-318**), and updated the text in **Discussion** to expand on our understanding and interpretation of the results (**l. 398-410**). We thank the editor and reviewers for the opportunity to clarify the interpretation of this analysis, since our own thoughts on this have evolved in response to the comments. We agree that the estimates from previous unsupervised, non-deep-learning methods are not as good of a “silver standard” as we believed. Those methods are not free from mis-specification (see also **l. 456-462** for a discussion we added on this topic, in response to point #1). In the updated manuscript we argue that the predictions by dadaSIA are likely to improve on previous estimates, where they differ from them. Our argument is based on the competitive performance of SIA with other leading methods in simulation-based benchmarking experiments, on the improvements from domain adaptation that we demonstrate here, and on the likelihood that the level of mis-specification pertaining to the well-studied CEU population is in a regime where domain adaptation should work well.

The additional points/suggestions raised by the reviewers are also important, and should also be addressed in a revision.

We thank all three reviewers for their insightful comments, which are often in close agreement with each other. We believe their suggestions strengthen the paper substantially. Another high-level change throughout the **Discussion** is we try to provide some guidelines based on results from our work for readers who might be interested in trying out domain adaptation with their own methods. We hope that this addition makes the manuscript more accessible to a wider audience.

# Reviewer #1

This study presents a careful application of domain adaptation techniques from comp sci to some example analyses in population genetics and provides a well written explanation of the approach. I view this as an important paper that I expect may benefit the field of pop gen, and it was very thought provoking. The main idea I took away, is the domain classifier branch with GRL *can only help* and should maybe be added to every neural network architecture; because, every empirical dataset is misspecified relative to the training set, and we don't know the full extent or quality of misspecification or else we could just simulate data that looks real (e.g. that includes background selection, accurate demography, etc). My biggest concern is whether, in some scenarios, tacking on the domain classifier branch to a network may actually hurt our inferences; this is important for guiding when and where to use domain adaptation. I reiterate this concern in the below list of comments and suggestions.

We thank reviewer #1 for their thorough and thoughtful comments. It was indeed our intention to make the argument that domain adaptation should be able to help in many if not most cases we are likely to encounter in realistic inference problems. But the reviewer raises an important point that the potential adopters of the method need some idea of the limitations to domain adaptation to decide whether it can help in their particular use case. We performed additional analyses of the efficacy of domain adaptation under different scenarios of mis-specification to probe its limit and the details on the changes are described in response to the below list of comments.

L75. "no way to ensure that the ranges considered are adequately large". I kept an eye out to see if you addressed "out of prior range" scenarios, but I don't think this was explored. Your misspecification scenarios were "qualitative" in that the training data lacked a piece of biological realism that was present in the test data. I'm curious about a more "quantitative" misspecification, where you try to estimate selection coefficients outside of the range shown in Figure 2. Or, for classifying sweeps, use test data that have very small or very large population sizes. I don't view this as totally necessary for your already useful analysis, but it seems like a potentially high reward simulation experiment.

This is indeed a very interesting experiment to perform. We have anticipated that domain adaptation would not work to “extrapolate” or predict out-of-range from training data. But we agree with the reviewer that it is worthwhile to test this conjecture directly. We now discuss this issue in **l. 439-449**, and present the result from the new experiments in **Fig. S5**. From our experiments, this does appear to be a problem that domain adaptation cannot solve, as we make clear to potential adopters in **l. 446-449**. We also discuss another method that addresses a related problem when the parameter of interest is sampled unevenly in the simulations (**l. 449-455**), which could be of interest to readers working on the broader problem of mis-specification.

I'm wondering about how to implement domain adaptation for other networks. (i) I was looking for explanation about how the "insertion point" is decided for the GRL/domain classifier branch? It seems to be an intermediate point in the network, but I can imagine this looking quite different for some other networks.

We believe the reviewer’s intuition is correct. In the **Methods** section, we added an explanation of how the “insertion point” is chosen for the CNNs implemented in this study, and suggested that the midpoint choice can be a good heuristic (**l. 471-478**).

(ii) How much target domain data is sufficient?

We have now tested the performance of the domain adaptive model with varying sizes of the target domain data. The results are presented in **Fig. S3C-E** and discussed in **l. 225-235**. In summary, the domain adaptive model performs best when the target domain size matches the source domain, retains reasonable performance when the target domain is a fraction of the source domain, but inevitably suffers when the target domain is only one tenth of the source domain.

(iii) The 20/80 experiment (and other proportions of sweep/no sweep) for the target domain was a great idea. In cases where we have some control over the target domain, e.g. exonic versus non-exonic regions, synonymous versus nonsynonymous snps, population 1 versus population 2—is it always better to balance the target domain?

This is a fascinating but difficult question to address in general. We believe that there are many factors that can be optimized depending on the particular use case, and that a full answer to this question is beyond the scope of the current manuscript. As we discuss further in our revised manuscript, there is much potential for future work to explore how domain-adaptive models can be further optimized for specific problems.

The 20/80 experiment was very useful. It did stand out to me that the 100% sweep case performed worse than the standard model. Although we shouldn't worry about 100% of human loci representing sweeps as in this particular experiment, I think this result is important to highlight in the main text, because it shows that domain adaptation is not necessarily >= the standard model. It makes me wonder what other weird test sets might cause the domain adapted model to perform not as well, and whether *my* empirical data are appropriate to use with domain adaptation.

We agree with the reviewer that this is an important caveat that should be presented to qualify our statement that domain adaptation *always* outperforms the standard model (this is by no means absolute). We now explicitly refer to this case in the **Results** (**l. 220-224**), but still believe that this type of scenario is rather unlikely in real applications.

Your simulation experiments are very convincing and cleanly implemented. I have tried hard to think of possible exceptions that might break things. I would expect the domain classifier to have no effect if the target domain was very similar to the source domain; but, is there any chance that training would suffer? I'm sort of thinking of GANs, how if the discriminator does poorly at the onset then the quality of training decreases. More likely, would it decrease prediction accuracy of the trained model slightly if the extra domain classifier branch was added, but not needed? Related, is there any chance that the added domain classifier branch of the network could be helping the network along simply by including additional layers and model parameters? Similarly, could the network be benefitting from showing it extra data, or the same source domain data twice, in a way that doesn't help it adapt the domain, but simply represents a bigger training set?

We thank the reviewer for this insightful comment. In an attempt to shed additional light on these issues, we ran additional experiments to test what happens to the domain adaptive model when there is no mis-specification. We found that the domain-adaptive model performs very similarly to the standard model when there is no mis-specification in the target domain, as if the domain classifier branch of the model does not exist (i.e. the domain classifier neither improves nor hurts performance). We present these results in **l. 281-289**, and explain this behavior in the **Discussion** (**l. 383-397**).

In the ReLERNN analysis, the GRL led to overcorrected predictions it seems. I thought this was an interesting "side effect" that might be discussed. Do you think that tuning the lambda parameter might alleviate this (although it seems hard to tune this with real data)?

From these results, it is not clear to us that overcorrection is occurring, but we do agree with the reviewer that the choice of λ is an important aspect of the domain adaptive model. We now discuss the optimization of λ as a future direction in **l. 503-507**.

L254. In the empirical results section, "these observations suggest that the addition of domain adaptation does not radically alter SIA’s predictions for real data but may in some cases improve them" (1) does not acknowledge the possibility that the results are worse than before, and (2) could be interpreted as "domain adaptation DID improve some estimates", but as you point out, we don't know the truth with these real data. And, the fact that the new results are dragged slightly towards the mean from the other methods that presumably do not account for background selection or demography does not support that the new approach is performing better, right? Unless you assume that Standard SIA performs poorly relative to the other methods? L309. Likewise, it doesn't seem like the evidence is strong enough here, unless you knew that the other methods are more accurate. I think it's perhaps interesting, reassuring in fact, that the results aren't so much different than Standard SIA in the empirical analysis. I was concerned however that SIA doesn't seem very accurate in Figure 2 even with Domain Adaptation. It seems to learn *some* signal for selection coefficient, but overall the predictions are quite noisy. If additional empirical analyses were performed I'd be curious to see maybe how the ReLeaRN results were affected. Like Relearn with and without domain adaptation applied to some real data (the paper did Drosophila I think?)

We thank the reviewer for giving us the opportunity to rethink and clarify the interpretation of the selection coefficient results. In part due to these comments, our thinking on this topic has evolved since the original submission. As discussed in the response to the editor, above, while it is not possible to arrive at a definitive answer in the absence of ground truth, we believe that dadaSIA provides improvement over previous methods based on their relative performances in simulation benchmarks and the likely degree of mis-specification present in the data (**l. 404-410**). We have removed the discussion on the directionality of the changes in the estimates that gave the unintended impression that unsupervised methods are better. Finally, we acknowledged the noisiness of the selection coefficient estimates, even with domain adaptation, and pointed out that there is room for further improvement for this somewhat challenging problem (**l. 398-400**).

I realize that the specific domain adaptation technique you use was described previously, but the technical description, and intuition behind the approach, seems like they could use additional explanation.

We agree that for readers who are interested in the method, the manuscript would be more accessible if the technical details are better explained. Accordingly, we expanded the descriptions in both the method summary in **Results** (**l. 133-145**) and the additional details in **Methods** (**l. 471-478, l.491-500**).

In your experiments, the "real" data are relatively similar to the "fake" training data, in the sense that they are both simulated with relatively simple models. However I was left wondering about the limits of domain adaptation. Real world empirical data have more than just background selection separating them from the training set, but also various other kinds of selection, intricate demographic histories (I know you looked at demography separately), population structure, and many other subtle factors that shape genetic variation. I think it's important to explore more complex scenarios, in order to answer the question: is there any potential downside to tacking the domain classifier, in practice? Does it ever contribute to additional error, if say, the real data are very very different from the simulated data. I suggest following up with an even more complicated misspecification scenario. For example one that includes BGS and demographic history simultaneously, the empirical ARG inference step as before, plus misspecified mutation rate, recombination rate, and gene conversion? Since releRNN seems to perform really well on the current simulations, perhaps they can be badly misspecified and some signal will still be retained. It might perform quite well on the badly misspecified data with the addition of domain adaptation! But ideally, I think, things should be pushed to the limit to understand how this works. Could also calculate summary statistics for each test set to quantify the divergence between source and target domains. I view this "badly misspecified" experiment as an important piece that will help guide when and if to use domain adaptation.

We agree with the reviewer (as well as the other reviewer and the editor) that probing the limits of domain adaptation would significantly strengthen the paper. To this end, we performed a series of experiments to test the performance of dadaSIA under increasingly severe mis-specification, up to a case where multiple aspects of mis-specification are present, following reviewer #1’s suggestion. We presented the result in the new **Fig. 5** and **l. 263-280**, and discussed them further in **l. 370-382**. We chose to explore this question in the context of SIA because of our familiarity with the model. In summary, we found, as expected, that the domain-adaptive model is robust to modest levels of mis-specification but inevitably breaks down when the level of mis-specification is extreme. As a result, we recommend that for any real-world application of simulation-based machine learning method, the simulation model should be constructed as realistically as possible, such that the remaining mis-specification likely falls in a range that domain adaptation can effectively address (**l. 380-382**).

## Minor points

Fig 1A. It could just be me, but I was unfamiliar with color-coded DNA alignment in the top left, didn't know what the colors meant.

This was indeed a mistake, we reused parts of a figure from a previous paper where the colors referred to non-recombining segments of the genome. The colors are irrelevant in the context of **Fig. 1A** and we have removed them.

I thought that SIA was an RNN, so I was confused about the conv/pool layers in figure 2 of the current paper?

The author is correct that the original SIA architecture was an RNN. We modified the architecture to a CNN because of the new genealogical feature representation used in this work. It was an oversight on our part that this update was not clear. We now explain the change and the reasoning behind it at the relevant **Results** section (**l. 162-167**).

L88. I agree that ABC methods are costly in the sense that extreme numbers of simulations are required to explore such vast parameter space; however there are nice approaches that deal with the many summary statistics, like the neural network regression from Blum and Francois (2010) and ABC with random forests (Pudlo et al 2016, Raynal et al 2019).

This was a mistake in writing. We actually wanted to point out that the number of parameters to be inferred is the challenge, not the number of summary statistics. We apologize for the confusion and have changed the text accordingly (**l. 86**).

L102. Is domain adaptation synonymous with generalization? I'm not certain but I thought the framing here might be "A variety of strategies for generalization have been introduced ... The specific kind we're interested in is domain adaptation".

From a brief survey of the literature, the term “generalization” seems to be used in different settings to refer to many distinct tasks in machine learning, some of which would include our application and some of which would not. Therefore, we prefer to stick to the narrower term “domain adaptation”, defined in the literature as specifically dealing with a target dataset that follows a different distribution from the training dataset. We clarify this in **l.90-94** in the **Introduction**.

L104. "domain adaptation can be incorporated directly into the process of training". It seems like the proceeding approaches also are incorporated in the training process. Maybe domain adaptation is distinct because it shows target-domain data to the network.

The reviewer is correct, other approaches also incorporate unlabeled target examples in the training process and this statement is inaccurate. We have removed it and rephrased the text (**l. 104-105**).

I for one could have used a brief description of precision-recall curves and the AUC metric. Maybe not necessary.

We appreciate the suggestion, but we think that these definitions will be clear to most reviewers and prefer to keep the manuscript lean. We will of course defer to the editor if further definitions are required.

Nothing is wrong with the contour plots (except, the dashed line is orange instead of yellow), but I personally like the scatter plots in the supp mat a whole lot better. Especially for the ReLeRNN results.

We updated the colors of the dashed line to match the contours (**Fig. 3B&D**), but after some further discussion, decided to keep the contour plots in the main paper because we think they are easier to interpret on a first glance.

L142. "reversing the gradient" I thought could use a bit more explanation at this stage, although I see you guide readers to the methods section. Maybe "reversing the sign of the gradient of the loss for the domain classifier for layers in the feature extractor"? Maybe not necessary, but it would have helped me if the architecture was explained more up front (and more clearly, see my below comments).

We agree that it is better to include a bit more detail upfront, and thank the reviewer for the specific suggestion on phrasing. We have paraphrased it and updated the text accordingly (**l. 141-143**).

L175. talks about the importance of the ARG inference step. It would have helped me follow along if a "(See methods section)" was added here. And also if the ARG inference step was mentioned in the figure 3 caption it would be helpful I think.

We added a new section in **Methods** that details the benchmarking experiment setups where we explain whether the ARG is inferred or not in each case (**l. 513-531**), which we refer to in the **Results** subsection (**l. 171-177**). In addition, we actually found the caption for **Fig. 1B** (**l. 753-756**) to be a good spot to emphasize that the target domain of SIA consists of inferred genealogies. We hope these modifications clarify the role of ARG inference in each benchmarking scenario.

L201. Mentions an alternative demographic model. But at this point, we don't know what the first demographic model used for SIA was.

We now clarified the setup for the SIA demographic mis-specification experiment in **l. 182-187**.

L248. Should "In all other cases" be "In all cases"?

We consider the estimate at LCT, which is one of the strongest signals (i.e. easiest case), to be highly consistent across all methods. The estimates all stay very close to 0.01. dadaSIA revised the estimates in all “other” cases that are not LCT. (Note that the line number is **l. 313** in the revised manuscript.)

L327. Confusing whether "GRL" refers to just one layer, or all layers in the domain classifier branch. I think it's just one layer, so it seems like this first sentence should instead say "we added a domain classifier branch with a GRL to the network." Seems like the domain classifier branch as a whole is insufficiently explained.

The reviewer is correct that GRL refers to a single layer. We have rephrased the description of the architecture to clarify the role of the domain classifier branch (**l. 471-474**).

L332. It's unclear if "output layer" refers to the output in the label predictor branch, the output in the domain classifier branch, or both?

Both, we have clarified this in the text (**l. 480-482**).

L380. peudo-real

This is a typo from a legacy version of the manuscript, which we have now fixed (**l. 571**).

L439. 1,111 windows... were 1000 SNPs sampled many times? How did it turn into 1,000,000 samples?

We sampled 1,000 SNPs from each of the 1,111 windows, yielding 1,111 x 1,000 ~ 1,000,000 samples. We have swapped the order of the clause to make this clearer (**l. 658**).

I would assume it's correct, but for the ASIP gene in the empirical analysis the previous estimate is 0.09 which seemed high.

We double checked the Stern et al. paper and this is indeed correct, albeit surprising.

# Reviewer #2

In this paper the authors present domain adaptive versions of two population genetics methods: SIA and ReLERNN. Since both methods (as well as most machine learning methods for population genetics) use simulated data for training, mismatches between training and testing/real data can cause issues in accuracy and interpretation of results. By incorporating a GRL (gradient reversal layer) component into the neural network architectures, the authors are able to mitigate the mismatch between the source (training) and target (testing) domains. The GRL part of the network is trained to identify source vs target examples, but then the gradient values wrt the weights are reversed, encouraging the overall network to use features common to both the source and target domains.

The authors apply both methods to "real" data simulated under different models from the training data, demonstrating improved inference results in the domain-adaptive versions. dadaSIA is also applied to real data (from European population CEU), although the results are a bit difficult to interpret.

Overall I love the ideas in this paper - the methodological advances could be widely applicable for other population genetics methods and could help us as a field understand what features of simulated training data are important for different applications. I have a few concerns with some of the details and a few suggestions for improving the manuscript.

We are grateful for Reviewer #2’s positive feedback as well as their thoughtful and constructive critiques that helped us significantly improve the manuscript.

## Major comments

1) The "simulation benchmark" (source-matched) and "hypothetical true model" (target-matched) experiments need to be described more clearly early on (starting around Line 163). I think this distinction is confusing to readers because when both the source and target datasets are simulated, the difference between "source-matched" and "target-matched" is not very meaningful for most methods (as you mention in Line 294). SIA is more of a special case where ARG inference is needed.

We now include a short description of the two “control” experiments in the opening paragraph of the SIA **Results** subsection (**l. 171-177**), with clear explanation of whether the genealogies are true or inferred in either case. For further details, we refer the readers to a new section in **Methods** titled “Setup of benchmarking experiments” that explains the four categories of experiments in detail and highlights the specific case of SIA regarding ARG inference (**l. 513-531**).

This is not a strong suggestion (as I would be open to reasons why not), but I would lean toward removing the "simulation benchmark" and just include the target-matched benchmark (as this is the desired case when the simulations match the "real" data). Further, I would perhaps suggest that the "standard" SIA model *not* include the true ARGs, but instead infer them in the same fashion as the real data. It feels unsatisfying to throw that information away from the simulations, but not doing so heightens the gap between the treatment of real and simulated data and makes it difficult to separate if the domain-adaptation modification is picking up on ARG inference issues vs. other differences between real/simulated data. Alternatively, you could try both (as is done in the original SIA paper I believe?)

We appreciate the suggestion, but we prefer to keep the “simulation benchmark” case to make the point that the common practice of using benchmark data simulated from the same model as the training data can lead to overly optimistic estimates of model performance (**l. 207-210**). We used the standard SIA model trained with true ARGs as baseline in order to demonstrate the versatility of domain adaptation to mitigate tree inference error in addition to mis-specifications. We agree with the reviewer that it is not clear in the current experiments whether the improvement came from one source or the other. To address this, we performed additional experiments where the only difference between the source and target domains is the tree inference step (“tree inference only” in **Fig. 5**). In short, we found that domain adaptation works to mitigate tree inference error either by itself or compounded with additional mis-specifications (discussed in **l. 363-366**). On a related note, we now discuss the pros and cons of domain-adaptation vs. building mis-specification/error into the simulations (**l. 431-438**).

2) SIA data representation. The ARG representation is quite different in this paper vs the original SIA paper. Was this choice related to the domain-adaptive version or just a more complete ARG representation? Does it improve results to use this representation in the standard model case? Overall I think this representation difference should be made a bit more clear throughout - as readers looking back at the original SIA paper may not be able to compare the results directly.

We now point out the updates to both input features and NN architecture of SIA in this study before we present the SIA results (**l. 162-167**) and refer the readers to **Methods** (**l. 601-623**) for details and rationale behind these changes. As we hope the new presentation makes clear, these changes are unrelated to domain adaptation. The reviewer is correct that the new feature encoding is a complete representation of the genealogies and should provide a baseline benefit for the original SIA model. We compared the new encoding to the original one empirically and showed that there is indeed a modest improvement (**Fig. S1C**). We also updated the original SIA codebase to utilize this improved encoding scheme.

3) CEU selection results. I think these should be reframed a bit (still included though). It is possible the dadaSIA results represents an improvement over SIA, but the number of examples is low and I am more struck by the differences between both SIA approaches and other literature. It would be interesting to investigate the causes of these differences (maybe more to do with general method differences?), perhaps in a future study.

We agree with the reviewer that we lack strong empirical evidence that definitively demonstrates the dadaSIA results are better. However, in the updated **Discussion** section, we try to present a more coherent argument that despite the lack of ground truth in real data, dadaSIA likely provides better results because the specific case of CEU population corresponds well to a regime where SIA is competitive against other methods in benchmarks, and domain adaptation should work to mitigate mild to moderate mis-specification in the simulations (**l. 404-410**). At the same time, we recognize the limitations of current methods (**l. 398-400**) and agree with the reviewer that more benchmark studies comparing selection inference methods are needed to explain their differences.

On this section - were the previously reported selection coefficients (from SIA) using the SIA method in [12] or the different ARG representation in this paper? If from [12] then it would be difficult to separate the difference due to the representation change vs the domain-adaptation modification, right?

The reviewer is correct that the previously reported estimates were from the original model and feature representation and changes to the SIA model must have inevitably contributed to the difference between the estimates by SIA and dadaSIA. However, as demonstrated in simulation experiments (**Fig. S1C**), we believe that the contribution from those changes is modest.

4) It seems like the demographic mis-specification for SIA (infer the demography from simulated "real" data) is not as extreme as for ReLERNN (where the source data has a constant population size and the target does not). Along with the ARG inference component, I think this makes it difficult to compare the results. The improvement for ReLERNN is not as large as for SIA (despite the bigger difference in source vs target). The authors highlight differences between SIA and ReLERNN in the Discussion, but I think it might be worth caveating the ReLERNN results a bit more - perhaps this method is more robust to simulation mis-specification than most.

The reviewer raises a good point that mis-specification might affect SIA and ReLERNN differently, especially considering they are designed for different tasks. We now explicitly point out the possibility that the standard ReLERNN model is more robust to mis-specification when comparing the results of the two methods (**l. 359-361**).

5) Interpretability. I think it would be very interesting to understand what the GRL is learning. Perhaps outside the scope of this study, but maybe the authors could include some discussion about how we might better understand gaps between simulated/real data through interpreting this component.

We are grateful that both reviewer #2 and #3 point out this important aspect of domain adaptation that we left out in the original manuscript. We now discuss interpretability in **l. 411-420** and believe that this will be of great interest to readers. Briefly, we plotted the loss curve of the domain classifier branch (**Fig. S9**) and found that it could be informative of the degree of mis-specification present between the domains. We acknowledge that this observation is preliminary and there is much potential for future work on model interpretation.

## Minor comments

-Line 59: "efficiently produce extremely large (virtually unlimited)" caveat this a bit... some selection simulations with large population sizes still very slow

We simplified this statement by just saying “large” (**l. 56**).

-Line 89: "scale poorly with the number of summary statistics" the bigger issue is (usually) that the simulations scale poorly with the number of *parameters* in the model. If summary stats are quadratic in the number of samples or number of sites, these can scale poorly too, but it's not usually the number of summary stats that's the problem.

We agree with the reviewer and have updated the text accordingly (**l. 86**).

-Line 103: "reweighting training instances" could you go into a bit more detail?

We expanded the sentence a bit (**l.101-102**), but the topic is complex so we think it is best to allow interested readers to refer to the cited papers.

-Line 150: "the demographic model used for the source domain was estimated from "real" data generated under a more complex demographic model and was therefore somewhat mis-specified"

From Fig S1A it seems like it's the same demographic model structure (just with inferred parameters) for SIA - maybe I missed something? (For ReLERNN it's more complex though.)

The reviewer is correct that the overall structure of the model remains the same, but the G-PhoCS model for inference is *under-parameterized*. We added some explanation in **l. 184-187** and refer the readers to **Methods** (**l. 558-563**) for details.

Methods: Could you include the # of trainable params/weights in each component?

We added these to the **Fig. 2** caption (**l. 766-770**).

Line 341: For training, is it the same source data for both components? I didn't quite understand if training is iterative (i.e. mixed source/target batch, update weights, then source batch, update weights etc) or not

In each epoch the same source data is fed through both branches, but the way the data is shuffled in mini-batches is different for each branch. In each mini batch training for the two branches is simultaneous, and the gradients from the two branches are combined to update the feature extractor weights. This is now explained in a bit more detail (**l. 496-500**).

Line 374: Maybe I missed this, but could you explain population A, B, C? This is from the out-of-Africa model right?

This demographic model is an Isolation-with-Migration model from a previous study on bird speciation (cited on **l. 555**).

Background selection section in Methods: (Line 357) "All mutations..." isn't there also a positively selected mutation for the "selection" examples?

That is correct. The beneficial mutation is separate from the deleterious ones. We have clarified this in text (**l. 542-546**).

## Editing

-Line 42: "the European" -> "a European"

-Line 123: missing "a"

-Line 173: "on it" -> rephrase

-Line 270: "ignored" -> "un-modeled"

We thank the reviewer for their attention to detail and we have made corrections following the suggestions above!

# Reviewer #3

In their study, Mo and Siepel show the impact of model mis-specification on the performance of simulation-based supervised deep learning methods in population genetics and propose an approach to mitigate the issue. More precisely, they rely on an adversarial domain-adaptation (DA) technique proposed in the machine learning field and integrate it into two different methods, (i) a selection inference tool SIA, previously published by their lab ; (ii) a recombination inference tool, RELERNN by Adrion and Kern. They convincingly show that domain adaptation can “rescue” a method that made otherwise poor and/or biased predictions.

Using two tools has multiple interests: (a) showing the versatility of the domain-adaptation technique that can be somehow “plugged” into different network architectures, for different tasks (estimating selection versus recombination), with different levels of expert knowledge regarding the original method (one is developed by their lab, one not) ; (b) investigating the effect of mis- specification and domain adaptation in different scenarios, where mis-specified “nuisance” parameters can have more or less impact on the performance ; (c) discuss the impact of tree reconstruction (used for SIA but not for RELERNN), but I will go back to this point in my comments.

Although I have multiple comments that I would like to see addressed for clarification or because I think they could help understand the applicability of the approach in certain conditions (eg little mis-specification, small target domain size), Mo and Siepel’s study is timely, thorough and overall well described. I thus think that, provided my comments are addressed, the manuscript should be accepted: it will be of wide interest to plos genetics readers, both methodology developers who could quickly try integrating DA into their tools, and users since it highlights clearly the impact of mis-specification (a general concern in the population genetics field) and explains the principle and additional value of DA in a way that should be understandable by many (with helpful figures).

We are grateful for reviewer #3’s encouraging words and their thoughtful and in-depth comments that help us substantially improve the manuscript. We particularly appreciate the suggestions for specific experiments that yielded very interesting results that help strengthen the narrative.

## Major comments

**Q1** The methodology underlying the “simulation benchmark” and “hypothetical model” should be stated more clearly. There might be confusion for the reader because it could be either (a) the original methods (SIA or RELERNN) trained and applied to the same dataset (in one case the “simplistic” simulations, in the other the hypothetical true - more complex – model); or (b) their domain adaptive version (with matching source and target domains). I understood it is (a), but it was not crystal clear at first read. Here is the sentence that I find ambiguous:

*l.161 For additional context, we also considered the two cases where the training and testing domains matched (source-matched or target-matched; Fig. 1C)—although we note that these cases are not achievable with real data and provide only hypothetical upper bounds on performance.*

Could the author clarify the sentence / the schematic table of Fig. 1 (add a short description in methods?)?

The reviewer is exactly right (it is *a*) and we appreciate the effort they put in to understand the exact experimental setup. To make it easier for readers, we have updated the schematic in **Fig. 1C**, expanded **Fig. 1** caption and provided a short summary of the two “control” experiments in the main text (**l. 171-177**). We refer the reader to a new section in **Methods** titled “Setup of benchmarking experiments” (**l. 513-531**) for additional details.

I actually see value in doing both experiments:

(a) Training and applying the regular method to matching domains has to be done since it provides the best possible performances (for simple simulations on one side, for hypothetical true model on the other). Compared to dadaSIA, it allows understanding if there is room for future improvements since, as the authors discussed l. 321, other domain adaptation techniques exist.

(b) Comparing (a) to the domain adaptive versions, still with matching domains, allows evaluating how much DA decreases (or not) the performances in a case where we would not have needed it. In this case, it can help understand the model’s behavior: Is the discriminator not learning anything, hence not influencing the computed features? Or is it somehow overfitting and giving unwanted feedback to the feature computation branch? The authors could also display training and validation losses of the different branches through training.

Of course, in real life it is complicated to know whether mis-specification is present (we can argue that it is very likely the case given that “all models are wrong”), and I agree with the statement that it is not achievable, so even if DA decreases performances in case (b), the method should still be valuable (but see Q2).

We thank the reviewer for the excellent suggestion and we have now tested the performance of the domain-adaptive ReLERNN model relative to the standard model when there is no mis-specification (i.e. DA is unnecessary). The result is summarized in **Fig. S7** and presented in **l. 281-289**. We plotted the loss curves in **Fig. S8** and discussed the observations and the implication in **l. 383-397**. In summary, our finding supports the reviewer’s first hypothesis – the domain classifier is not learning anything when there is no mis-specification, does not contribute to training the feature extractor, and therefore the model behaves very similarly to the standard model.

**Q2** Related to Q1, if one observes a decrease in performances in (b), it would be helpful to understand what level of mis-specification is “required” for DA to start being valuable (i.e. to start increasing performance compared to the non DA standard application case). To design such an experiment, one needs target datasets that get increasingly divergent from the source with small incremental steps. For example, it could be the background selection experiment with a purifying selection coefficient going gradually away from zero. I am aware that it is a costly experiment as it requires a new training for each step, but I believe the answer informative to know to what extent researchers should all move to using DA in neural networks, or if this has a hidden risk.

This is an excellent suggestion. We opted to address it using the demographic model rather than background selection, by parameterizing the degree of mis-specification using the size of a bottleneck (**Fig. S4**). The suggestion of different strengths of background selection could also be informative, but it is more complex to carry out—the DFE involves several parameters whose impact on mis-specification is more subtle. It could be an interesting experiment for future work.

We added a new **Fig. 5** and a section titled “Efficacy of Domain Adaptation under Various Degrees of Simulation Mis-specification” to the main text (**l. 263-280**) that summarizes the result from the experiment. Briefly, we found that DA works well with mild to moderate mis-specification. However, as might be expected, DA eventually fails when mis-spec becomes severe. Therefore, in real-world applications, we should be concerned with cases of extreme mis-specification and try to avoid them by optimizing the simulation model to the best of our ability before applying domain adaptation (**l. 380-382**).

**Q3** The target domain size is currently the same as the training size, i.e. very large. I am not certain it is a reasonable assumption, given that the target domain corresponds to the data, which in many cases is more limited than simulations. For example, in the real application of SIA the target size is 1 million; however, those are not independent examples (contrary to the simulated target domains). ReLERNN was not applied to real data, but I understand that we would expect to have fewer target examples, since one example does not consist of a single tree but of a long genotype matrix. So would DA work as it is currently set up? This could influence the strategy chosen for the loss weight (l.347-348).

The authors should comment on those points and, if they agree that size is often limited for real data, evaluate the impact of source-to-target size ratio / discuss based on previous ML literature, as this might limit the use of DA in practice.

I note that the authors provide an interesting related experiment where the labels are imbalanced in the target domain but not in the source domain. It is also very relevant in terms of benchmarking for a realistic target domain, hence for applications to real data, but answers a different question.

We agree with the reviewer that the user has little control over the target domain and therefore these assumptions of an ideal target domain may not hold true. We tested the performance of the domain-adaptive model when the target domain size is limited and presented the result alongside the imbalanced label experiments in **Fig. S3** and **l. 225-235**. Briefly, we found that while there is some tolerance for a smaller target domain, the performance of the domain-adaptive model decreases when there are fewer target examples. Nevertheless, the domain-adaptive model still improves upon the standard model when the size of the target domain is only 10% of the source domain.

**Q4** The discussion around the impact of tree reconstruction versus other nuisance parameters (demography/background selection) is interesting, especially given the recent trend of relying more and more on reconstructed trees and extending tree inference tools to other tasks. However, I am not fully convinced that the following statement is completely backed up:

l.285 *For example, the performance gap between the “simulation benchmark” (trained and tested on simulated data) and “hypothetical true” (trained and tested on real data) models was considerably greater for SIA than for ReLERNN (Figs. S2C&D, S4C&D). This difference appears to be driven by ARG inference, which is required by SIA in the hypothetical true case but not the simulation benchmark case, and for which no analog exists for ReLERNN.*

Indeed it is possible that the demography and selection mis-specifications have way more impact on SIA than on RELERNN, regardless of tree reconstruction, leading to the qualitative differences in the usefulness of DA for the two tasks.

A perfect experiment would be to include NO mis-specification for these nuisance parameters (demo or selection) but only for the tree reconstruction (source with true trees, target with reconstructed trees) and compare performances without and with DA. Was this done? If yes, please clarify the text. If not, the authors should temper their conclusion or, more interestingly, do the experiment. This experiment would be useful to many researchers developing inference tools based on reconstructed ARGs.

This is a straightforward yet illuminating experiment to do and we thank the reviewer for the suggestion. The result is presented as the ”tree inference only” case in **Fig. 5**, and it shows empirically that DA does indeed mitigate mis-specification due to tree reconstruction error alone (**l. 364-366**).

Going further, but likely for future work, one wonders if DA is better to fix the gap than reconstructing the trees for all simulated data so that both source and real target domain are based on inferred trees. Even if slightly worse, the computational gain of using DA and not inferring all trees could be valuable.

We thank the reviewer for pointing out the computational advantages of DA vs. tree inference. We now discuss the pros and cons of two alternative strategies of building error into labeled training data vs. performing domain adaptation (**l. 431-438**), and agree with the reviewer on the need for future benchmark studies.

*Important: The discussion could be extended to reply to the following questions Q5-8*

**Q5** Could the authors discuss the impact of prior mis-specification for target parameters (such as real selection coefficient or recombination rate out of range) rather than for nuisance parameters (demo etc). I foresee that DA will fail in such a case.

We also expected DA to fail in these out-of-range inference tasks and now have demonstrated that it is indeed the case with a simulation experiment (**Fig. S5**). An informal analogy is that one cannot expect a ML model trained with only examples of digits 0-4 to recognize digits 5-9. We also compare this scenario of *range* mis-specification to a related case of *distribution* mis-specification (**l. 439-455**) and hope that the readers will find this interesting.

**Q6** Model mis-specification also strongly impacts non deep learning approaches (such as ABC on summary statistics) and even non-simulation-based approaches (model-based likelihood with simplifying assumptions). It could be good to discuss this, and I would enjoy reading the authors' view on whether DL approaches are the only ones that could benefit from such domain adaptation techniques.

We thank reviewer #3 as well as the editor for giving us the opportunity to offer our thoughts on this topic and discuss our work in a greater context. We agree that mis-specification is almost omnipresent in real-world applications. In **Discussion** (**l. 456-468**), we broadly categorized mis-specification into that which concerns *simulations* in a supervised ML setting (simulation mis-spec) and that which concerns *model* choice in a likelihood-based setting (model mis-spec). We argue that the simulation mis-specification problem, in a sense, allows for more straightforward empirical solutions such as domain adaptation. By contrast, the model mis-specification problem typically requires more difficult and labor-intensive customized analyses for identification and correction.

**Q7** Although many previous works discussed model mis-specification and its impact on their methods by setting up robustness experiments (to different demographic models, to selection instead of neutrality, etc.) extremely few tested a solution, which is why this study is very valuable. One could discuss the papers that intentionally widen the models/priors to integrate more nuisance parameters, simulate errors in the data, or aim at co-estimating multiple processes (e.g. demo + selection). But the work closest to this study in terms of mis-specification mitigation is the one on automated inference using gan (cited by the authors). Hence it could be good to discuss a few resemblances and differences / advantages between the approaches. Some are clear to me (for example, the current approach is very flexible and integrable to most of the existing neural networks for population genetics inference, and likely lighter computationally – but see a question below) but maybe less clear to a regular reader. I might also be missing some disadvantages.

We appreciate the reviewer’s confidence in our work. We do think that domain adaptation is very flexible and scalable, but can be difficult to interpret (although there is definitely room for future work on this front). We now compare the main strategies to address simulation mis-specification in **l. 431-438**.

**Q8** In their previous paper (SIA Hejase et al. 2022), the authors did multiple experiments to test the impact of ARG inference and demography mis-specification. Could the authors put them in perspective with the experiment and findings of the current study? For example: (i) Are the levels of mis-specification and the yielded estimation errors similar between the two studies? (ii) Although the classification is reasonable, the coefficient estimates of SIA standard application (Figures 3&S2) are really poor (ie they do not seem usable in practice). It is a bit worrisome for papers using the original tool, as the mis-specification scenarios are very realistic. Can Hejase et al (2022) provide insight on how CLUES and imagene or other methods would perform in these scenarios?

The mis-specification experiments from the original SIA publication (Hejase et al. 2022) and those in this study are not directly comparable because the exact setup of simulation mis-specification is different. In this work, we examined the presence of background selection as well as different types of demographic mis-specification. Hejase et al. focused mostly on mild to moderate mis-specification in population sizes. In addition, the original SIA model employed an *ad hoc* strategy to deal with mis-specification caused by ARG inference. Despite the availability of true trees, the model was trained with genealogies inferred from the simulated sequences in hopes that it learns to take inference error into account when making predictions (now explicitly discussed in **l. 433-435**). Therefore, the performance of the “standard model” in this study (trained with true trees, applied to inferred trees) is likely only a lower bound of that of the original SIA model. To make this clear, we updated the terminology in **Table 1** and added a footnote to highlight this difference. Ultimately, we believe that for future work it will be valuable to benchmark the performance of different methods under a systematic set of mis-specified scenarios and also compare different strategies to mitigate mis-specification under those scenarios.

## Other comments

- Are the comparisons always done using the improved input features (cf Fig S1)? Otherwise, I am worried that differences might not reflect (only) the introduction of DA but also the change of input representation. This question applies to benchmark experiments and the real data application (Table 1).

The benchmark experiments in this study always use the improved features and NN architecture, for both the standard and domain-adaptive models. We now make this clear in **l. 162-167**. In **Table 1** we took the previously published value from the SIA paper (Hejase et al. 2022) which came from the original SIA model. It is likely that the updated feature and model led to some improvements, but the contribution seems to be modest (as shown in **Fig. S1C**).

- What is the cost of training domain adaptive networks? How does it compare to the original versions?

We now discuss this in **Methods** (**l. 507-512**). The computational cost for DA models is on par with the original versions.

- It would be great to give, in Sup Mat, training and validation losses (decomposed along branches) to get an insight on the model training behavior.

We show this in **Figs. S8&S9**, and discuss them in the context of model behavior when there is no mis-specification (**l. 387-397**) and estimating the degree of mis-specification using domain classifier loss (**l. 414-416**). We thank the reviewer for this suggestion that turned out to be quite insightful!

- Related, is there a way to leverage the gradient or losses information to approximate how mis- specified a model was? Losses with adversarial training can get tricky to evaluate, so this might not be trivial.

As mentioned above, we added **l. 414-416** in **Discussion** to present the preliminary observation that the domain classifier tends to start with lower loss when mis-specification is more severe. We believe that there is potential for future work to leverage this information to unpack what the domain classifier is learning (or “un-learning”) (**l. 416-420**).

- l. 89 except maybe for ABC-RF

As mentioned previously in response to the other reviewers, we realized that the number of sum stats is not the problem. We actually wanted to point out that ABC scales poorly with the number of parameters to be inferred. We have rephrased the text accordingly (**l. 86**).

- Fig2 in the original SIA paper, the architecture is depicted as an RNN (LSTM) with no mention of convolutions, here there are only mentions of convolutional blocks. Please clarify whether some changes were made.

The reviewer is right that we did not explicitly mention the changes we made to the SIA model in this study. We now point out these changes in the opening paragraphs of the SIA result sections (**l. 162-167**) and refer the readers to the **Methods** for rationale behind these changes (**l. 614-623**).

- l. 150- 153: it's a good way of setting the mis-specification. It would be useful to summarize in the text the main resemblances/differences: ie the model still consists of the same number of populations with constant population size in between split events and migration only between B and C, but the split between B and C is older followed by higher rates of migration compared to the source model.

We reiterated this setup in the SIA specific **Results** section (**l. 182-187**) and refer the readers to the **Methods** for descriptions of the main differences we added (**l. 558-563**).

*- l. 178 “In our experiments, the ARG is “known” (fixed in simulation) in this case, whereas in the hypothetical true model it must be inferred. Thus, the difference between these two cases represents a rough measure of the importance of ARG inference error (see Discussion).”* and l.285 *For example, the performance gap between the “simulation benchmark” (trained and tested on simulated data) and “hypothetical true” (trained and tested on real data) models was considerably greater for SIA than for ReLERNN (Figs. S2C&D, S4C&D). This difference appears to be driven by ARG inference, which is required by SIA in the hypothetical true case but not the simulation benchmark case, and for which no analog exists for ReLERNN.*

I understand that (for SIA) for simple simulations, both source and target the true tree is used, and for hypothetical true, both source and target, the tree is inferred, whatever the experiment. Can the author clarify this in l178 and Method. It would **be very helpful** to have the equivalent of the schematic table of figure 1 for the SIA case, where the authors would add in each cell if trees are exact or inferred (possibly in sup mat).

I think that when the trees are inferred in both source and target, it is not a case model mis- specification but corresponds instead to noisy input data. Alternatively, when the true trees are used for the source and the inferred ones for the target, I agree it can be depicted as a type of model mis- specification.

The **source** is actually *always* **true** genealogies and the **target** is *always* **inferred** genealogies. It’s just that for simulation benchmark the model is both *trained* and *tested* on source (i.e. source-matched) and for hypothetic true the model is both *trained* and *tested* on target (i.e. target-matched). This is illustrated in **Fig. 1C**. To make this clearer, we modified **Fig. 1B** to highlight the words “true” and “inferred”, as well as the figure caption to reiterate the distinction. We added a new section in **Methods** to further explain the details of our benchmarking setup (**l. 513-531**).

- l.187 Imbalanced classes experiment. Can the author clarify whether the error is computed on a balanced test set or on a similarly imbalanced test set for each percentage? Could it also make sense to show the standard model error on each differently balanced test set?

We clarified in **Fig. S3** caption (**l. 690-691**) that the performance is always evaluated on a balanced test set. It could be a good idea to evaluate on differently balanced test sets, but it seems that Precision-Recall curves are robust to imbalance in the test data (which is why we picked them over ROC curves).

What is the expected imbalance in, say, humans and other well-studied species?

This is a great question but we are not sure. We believe that there might be a wide range depending on the specific population of interest. Addressing this question likely requires domain knowledge from users of the method.

- l.298 Fig 4,S4: Do the authors have an intuition regarding the underestimation of high recombination rates without DA, and the underestimation of low rates with DA?

Perhaps the reduced haplotype diversity due to a bottleneck or purifying selection results in lower estimates of the recombination rate to the model without DA. We are less sure about underestimation of low rates with DA. We regard this topic as too speculative to discuss without further experiments.

- l. 241: *The seven loci predicted by SIA to be sweeps were also predicted by dadaSIA to be sweeps (Table 1), although dadaSIA always reported higher confidence in these predictions (...)*

Was this phenomenon of increased confidence observed in the previous experiments?

We are not sure what “previous experiments” refer to. Selection inference in the 1000 Genomes CEU population presented in this paper is the first time we applied dadaSIA to real data, therefore it remains to be tested empirically in future studies whether dadaSIA infers sweeps with higher confidence.

*- l.254 Together, these observations suggest that the addition of domain adaptation does not radically alter SIA’s predictions for real data but* ***may in some cases improve them.***

The last part is a bit of a gamble because SIA was previously shown to be better than other methods (although I do not know if it was compared to all the methods used for the last column of Table 1), so going away from SIA estimates towards the literature’s estimates might not be a good thing. Unless SIA with mis-specification is worst than previous methods? I believe the authors do not discuss this.

SIA was indeed previously shown to be competitive with other methods in terms of robustness to mis-specification (Hejase et al. 2022). Our thinking on the empirical results has evolved since the initial submission and we thank the reviewer for the opportunity to clarify this. The estimates from previous unsupervised, non-deep-learning methods are not as good of a “silver standard” as we initially believed. We updated the **Discussion** to reaffirm that we believe the dadaSIA predictions likely improve on previous predictions because the CEU population corresponds to a regime where dadaSIA is likely to be the most effective (**l. 404-410**).

- l. 343 and 345: State the exact losses (MSE and binary cross entropy ? Penalization ?)

We added the losses in **l. 493-494**, **l.496**.

- l.349-361: It is not clear from this paragraph whether trees are inferred in some cases of the background selection experiment. It seems that not, but l.178 seems to say otherwise. Can you clarify? Or is it that l. 374-383 correspond to a methodology that applies to both types of mis- specification and should have its own title? Then l 384-390 rather fit in the “Demography mis- specification experiment with SIA” part, since demography inference is not done for the background selection experiment? Please clarify.

We apologize for the confusion. We swapped the order of **Methods** sections without updating the text. The trees are indeed inferred for the background selection experiments as well and we clarify in **l. 546-547**. Now the two separate sections on background selection and demography mis-spec should each be self-contained.

- in l 374-383. Can the author specify whether only the central tree of each simulated segment for pop B will be encoded and used to train SIA and dadaSIA for readers unfamiliar with SIA?

The reviewer is correct and we clarified this in **l.575-577**.

- l.388 I did not find the G-PhoCS file

We added the control file on Aug 31, 2023 at github.com/ziyimo/popgen-dom-adapt/blob/main/manuscript_aux_files/GPhoCS_ctl/deminf_3pop.ctl.

- Method: The author should provide their code for the different simulation scenarios

We updated the GitHub repo on Aug 31, 2023. The simulation scripts are at github.com/ziyimo/popgen-dom-adapt/tree/main/manuscript_aux_files/sims.

- l.420 indicates the split training/validation/test for RELERNN; is it similar for SIA?

We clarified the exact split in each relevant method section (**l. 548, 574, 593**).
