# Supplementary material for: Domain-adaptive neural networks improve supervised machine learning based on simulated population genetic data
Source: PLoS Genet. 2023 Nov 7;19(11):e1011032. doi: 10.1371/journal.pgen.1011032 (PMC10655966; doi:10.1371/journal.pgen.1011032)
Supplement: Attachment — Submitted filename: response-to-comments-rev2.docx [file pgen.1011032.s012.docx]

**Editor’s comments**

The reviewers and I were generally pleased with the revisions and are enthusiastic about the paper. There are just a few outstanding minor comments where some additional change/clarification in the text is necessary.

We thank the editor and reviewers for their continued interest in our work! We are happy to clarify the additional questions raised by the reviewers and in the following responses describe the changes made to the manuscript.

**Comments to the Authors**

**Reviewer #1:** The authors sufficiently addressed all of my comments and questions. They also did substantial simulation experiments exploring the limits of the technique, which I appreciate, and the revised presentation seems effective and fair. I recommending accepting this great paper.

We are grateful for the reviewer’s positive feedback.

**Reviewer #2:** The authors have completed a substantial revision of their original paper. In particular the new analysis of varying levels of mis-specification is very helpful, along with the inclusion of loss curves for the different components of the network. They have also clarified the real data CEU results and generally improved the manuscript to make many aspects clearer. My initial comments have all been addressed, including the differences (in data encoding and architecture) between SIA and dadaSIA which are clear and consistent now. I just have a few questions about the loss analysis, in particular this interesting observation:

We believe the idea of loss analysis suggested in the reviewer’s initial comments led to results that readers will find really interesting, and we thank the reviewer for the chance to further clarify and interpret the observations.

*"We observed that the domain classifier generally tended to start with a lower loss and took more epochs to train when the mis-specification is more severe (Fig. S9). It might be worthwhile, as a future endeavor, to try to identify the features driving this loss, understand their evolutionary significance, and, perhaps, incorporate them into a new set of simulations. In such a way, domain adaptation could be used to discover evolutionary processes and improve the models used for simulation."*

The idea of a lower loss when there is more mis-specification make sense to me. But when I was thinking about the domain-classifier loss trajectory (and also went back to the original domain-adaptive paper [30]) it seems like we are still trying to *minimize* the domain-classifier loss (i.e. learn the difference between the source and target domains) right? But due to gradient-reversal in the feature-extractor, this becomes difficult over training, so the loss increases?

We are actually trying to ***maximize*** the domain-classifier loss (***un***-learn the difference between the source and target domains, hence achieving a “domain-invariant” representation of the data). As the reviewer mentioned, the gradient-reversal layer is designed specifically to accomplish this. This is also described on l. 11, page 2 from [30] (<https://proceedings.mlr.press/v37/ganin15.pdf>). In other words, the increase in loss is a “feature” of the GRL, not a “bug”. We now make this clear in **l. 488-489**.

I guess I expected to see that as mis-specification increases, the loss would be somewhat low even at the end of training (i.e. still easy to distinguish source and target). I was surprised to see that in Fig S9 for example, all models eventually achieved random guessing (albeit with the "extreme" case happening more slowly).

This is indeed a very interesting observation and reminds us of the following quote:

*''One may go wrong in many different ways but right only in one, which is why it is easy to fail and difficult to succeed—easy to miss the target and difficult to hit it.''*

Our intuition is that in the optimization landscape of the (very high-dimensional) parameter space of the domain classifier, there are only a few regions where the domain classifier does a good job distinguishing the domains, by picking on the correct features specific to each domain. However, there are vast regions where the domain classifier can do badly, by avoiding the domain-specific features. Therefore, it is perhaps not hard for the model to arrive at the “bad” regime during training.

We refrain from discussing this in the manuscript so as not to make the manuscript too verbose.

I was also surprised that the loss was almost 0 (i.e. no classification errors) right away. Maybe for the more extreme mis-specifications it's an easy classification problem, but it seems like it would take at least a bit of time to learn the difference.

The reviewer is correct that the domain-classifier losses were very low at the end of the 1^st^ epoch. So far, the model had seen all training data once, and the feature extractor has already been trained jointly to perform the label prediction and to *not* distinguish the domain. Perhaps at this point the model picked up features that are useful for label prediction that also happens to be useful for domain classification (hence the low domain-classifier loss), and it takes additional epochs to *un*-learn those features.

I think I'm missing something and that's leading to my confusion - if the authors could clarify the loss results a bit more that would be very helpful!

It is clear that the reviewer has put a lot of thought into the inner-workings of how and what the domain-adaptive model learns during training and we really appreciate it. There is definitely room for future work to further investigate these fascinating questions.

**Reviewer #3:** I am very positive about the revision of the manuscript. The authors made a substantial amount of experiments to address my comments and clarified the unclear sections. I read with interest the outcome of these new experiments and I am happy to see that they confirm the intuitions of the authors, reviewers and editor. It is a good news that the domain adaptation does not decrease the performance where there is little or no mis-specification, i.e. that the classifier was not weirdly overfitting. It is also a good news that even with little target data (1:10 of the source size) the performance remain similar or better than the standard model, making me more confident that DA is suitable in practice. Finally, I was glad to see that losses gave some insights on mis-specification levels and on the inner working of the network. Overall, I am very happy with the authors’ work and, as I had already said, convince of the study relevance.

We are glad that the reviewer found the results of the new experiments insightful and we again appreciate the thoughtful comments from the editor and reviewers that inspired these experiments.

Minor comments:

l.458 I would not classify ABC as an unsupervised method since the simulation set has labels that are used for inferring the posterior. ABC with correction steps are even learning a supervised machine learning model (penalized regression, dense neural network, ..) locally (in the region of the observed data)

We thank the reviewer for pointing out this important distinction and we have removed ABC from the list of unsupervised method (**l. 458-459**).

- The authors said they were not sure what I was referring to in one of my comment
Me: Was this phenomenon of increased confidence observed in the previous experiments?
Them: “We are not sure what “previous experiments” refer to.”
What I meant is that the experiments to benchmark the dadaSIA are commented in terms of RMSE, AUPRC etc. Not in terms of prediction confidence. The authors observed and increase of confidence of dadaSIA compared to SIA for real data application (l.308, 403). Did they also observe a change in confidence between SIA and dadaSIA when applied to simulations?
It might be another advantage of domain adaptation if confidence appears to be well calibrated. (But can be kept for future study if the answer is not obvious).

Thank you for clarifying this comment!

Intuitively, the better precision-recall performance (**Fig. 3A&C**) in general should translate to increased confidence in model prediction. P-R curves can be considered representing how well the model is "separating" two classes in the probability space. If the P-R score is high, the classes should separate well in the probability space, and the confidence should be high (higher probability for positive class, lower probability for negative class), whereas if P-R score is low, the classes are not separable and the probabilities are mushed together, which translate to lower confidence in prediction.

However, we do not discuss this in the manuscript since the reasoning is a bit circuitous and we agree with the reviewer that future work can help address this question directly.
